# Effect of social support and health education on depression scale scores of chronic stroke patients

**DOI:** 10.1097/MD.0000000000017667

**Published:** 2019-11-01

**Authors:** Fu-Huang Lin, Daphne Ng Yih, Feng-Mei Shih, Chi-Ming Chu

**Affiliations:** a School of Public Health, National Defense Medical Center; b School of Occupational Therapy, College of Medicine, National Taiwan University; c Department of Rehabilitation Medicine, Tri-Service General Hospital, National Defense Medical Center, Taipei; d Department of Healthcare Administration and Medical Informatics, College of Health Sciences, Kaohsiung Medical University, Kaohsiung City; e Department of Medical Research, Kaohsiung Medical University Hospital, Kaohsiung; f Big Data Research Center, Fu-Jen Catholic University, New Taipei City, Taiwan.

**Keywords:** leisure activity, post-stroke depression (PSD), social support

## Abstract

Post-stroke depression (PSD) constitutes an important complication of stroke, leading to great disability. After stroke, the prevalence rate of depression is about 30%. Depression also affects rehabilitation motivation, delays function recovery, and increases family and social burden. The objective of this study was to explore the effect of social support on depression in chronic stroke patients and the relationship between demographic and disease characteristics. Total samples were randomly divided into an intervention group (n = 31) and a control group (n = 31). Sixteen social support interventions were performed over 8 weeks. Social support programs were implemented 2 times a week. Depressive symptoms were assessed at the second week, 4th week, 8th week, and 4 weeks after the end of the study using the 10-item Center for the Epidemiological Studies of Depression Short Form (CES-D10). There was a significant correlation between depression and the economic status of the patients with chronic stroke, satisfaction in leisure, the presence or absence of caregivers, the duration of stroke, and with or without pain. A significant difference was found between two groups after social support for 8 weeks. Our findings suggest that remission of PSD needs at least 8 weeks of social support.

## 1 Introduction

Stroke is the third leading cause of death in Taiwan. After an acute stage, stroke patients usually suffer from physical, mental, verbal and social function disorders in varying degrees; in particular, post-stroke depression (PSD) is not only an important sequela, but also an important factor to predict the quality of life. Epidemiological studies have shown that about 30% of stroke patients at early or late stages develop PSD, which affects rehabilitation motivation of the patients, reduce the rehabilitation effect, and increase the load of family care. Although PSD affects the quality of life and functional recovery, it is often overlooked.^[1]^ According to statistical data, the prevalence of depression is about 29% within 10 years after stroke, and the 5-year cumulative incidence is about 39% to 52%.^[2]^ In a study on PSD and post-stroke fatigue of 368 stroke patients hospitalized within 3 months, researchers found that brain damage could result in physiological and psychological impairments. To be able to live independently, patients should learn the skills of adaptation, including the ability to seek social resources.^[3]^ Successful rehabilitation means patients are able to maintain original social relations and actively participate in social activities to return to community life. Community social interaction or participation in activities requires physical and psychological ability.^[4]^ In addition to rehabilitation activities, functional therapists should also meet the psychological needs of stroke patients in order to achieve holistic health care. In this study, we have investigated the effects of routine rehabilitation activities and additional social support and health education by functional therapists on PSD, and proposed suggestions on home and rehabilitation-related activities.^[5,6]^

## 2 Materials and methods

### 2.1 Research design and subjects

**Figure 1:**
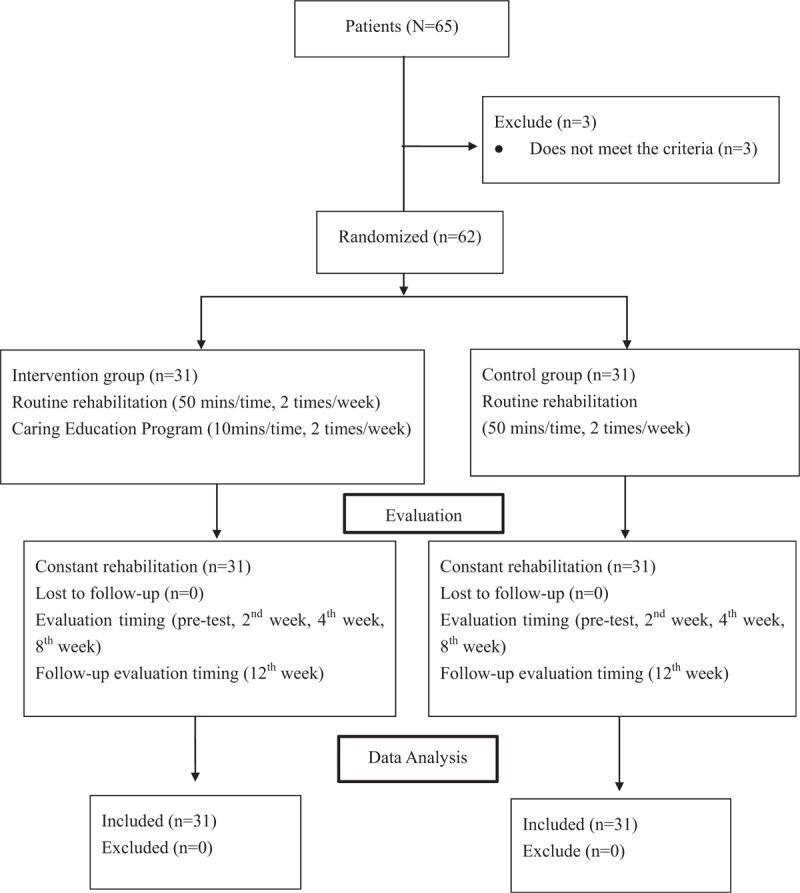
Flow chart of the enrollment.

This study was an interventional study, and subjects were stroke outpatients who visited a rehabilitation department between October 2010 and February 2017. Inclusion criteria were, people who had suffered the effects of stroke for up to 6 months (since first onset) and returned to their community without recurrent stroke within half a year. Exclusion criteria were people with depression, impaired cognition (Mini-Mental State Examination, MMSE < 20), or language disorder (Fig. 1). Sixty-five patients were recruited, but 3 patients were excluded due to impaired cognition and serious language disorder. The remaining 62 eligible patients were randomly assigned to an intervention group and a control group. After intervention measures were taken, changes in depression scale scores before and after treatment were compared between the 2 groups. This study was approved by the ethical committee of the Institutional Review Board of Tri-Service General Hospital in Taipei City, Taiwan (TSGHIRB No. 2-105-05-036).

### 2.2 Research tools

#### 2.2.1 Structured questionnaire

Demographic and stroke characteristics data were collected. Demographic data include gender, age, education, economic status, living conditions, caregivers, and leisure satisfaction; stroke disease data include stroke duration (calculated from the date of first onset), daily life function, type of stroke, number of stroke, dominant side, pain, and cognitive score.

#### 2.2.2 Center for Epidemiologic Studies Depression Scale (CES-D)

A depression scale (30 scores in total, the higher the score, the more severe) was used which included ten questions from the “Taiwan Longitudinal Study on Aging” by the Health Promotion Administration, Ministry of Health and Welfare. Cut-off scores in order of depression severity were based on other related studies in Taiwan.^[7,8]^ In the present study, 8, 9, and, 10 scores used as cut-off scores to discuss depression tendency were evaluated in the 1st week (before intervention), 2nd week, 4th week, 8th week, and 12th week.

### 2.3 Intervention measures

According to Cohen et al, social support can be divided into 4 categories:

(1) emotional support such as caring and empathy provided by relatives, friends or persons of significance;
(2) informational support such as required knowledge and advice;
(3) instrumental support such as materials, money, or other resources or assistance; and
(4) friendship and social interaction.^[9]^

In the present study, social support and health education include:

(1) rehabilitation-related informational support, such as providing consultation on rehabilitation at home after stroke and solving life problems;
(2) emotional support, including caring, encouragement, and empathy, such as advice for enjoying leisure time at home or dealing with inner anxiety.

An execution frequency was set by reference to other related studies in Taiwan.^[10]^ In the intervention group, routine rehabilitation activities were provided twice a week, 50 minutes at a time, plus 10 minutes of social support and health education at a time. In the control group, only routine rehabilitation activities were provided twice a week, 50 minutes at a time, without any other intervention. Rehabilitation activities were provided 16 times over 8 weeks (1st–8th week); there was no intervention from the 9th to the 12th week. Final assessment was conducted in the 12th week.

### 2.4 Statistical methods

The demographic characteristics and stroke features and depression scores are described by the number of cases or by means with standard deviation. Mann–Whitney *U* Test was used to examine the differences in the general demographic characteristics and intervention measures between the intervention and control group. In addition, the changes of the depression status in intervention were tested by Wilcoxon signed rank test and McNemar test. The relationship between variation in depression scale index and time effect were measured using generalized estimating equations (GEE).

Statistical analyses were conducted using SPSS version 22.0 (SPSS, Chicago, IL). All tests were 2-sided, and *P* values of less than .05 were considered statistically significant. This study considered a *P* value of less than .05 as significant for all analyses.

## 3 Results

There were 62 eligible subjects (mean age: 63 ± 13.8 years) in this study, including 44 males and 18 females. Thirty-one subjects were placed in the intervention group (mean age: 65.1 ± 9.19 years); 21 males and 10 females. Another 31 subjects were placed the control group (mean age: 61.1 ± 13.8 years), including 23 males and 8 females. Among the 2 groups, the mean duration of stroke was 73.1 ± 51 months. In the intervention group, the mean duration of stroke was 65.1 ± 51.8 months; in the control group, the mean duration of stroke was 80.9 ± 48.9 months. Before treatment, the mean depression score in the intervention group (7.4 ± 6.9 scores) was higher than that in the control group (5.1 ± 4.5 scores); in terms of the type of stroke, 22 patients had infarcted stroke and 9 patients had hemorrhagic stroke in either group. In terms of number of times of stroke onset, 25 patients had experienced 1 onset of stroke and 6 patients experienced more than one onset of stroke in the intervention group; 22 patients had experienced 1 onset of stroke and 9 patients had experienced more than 1 onset of stroke in the control group. Thirty-five patients had dominant-side limbs affected and 26 patients had non-dominant-side limbs affected. Among them, 19 patients in the intervention group and 16 patients in the control group had dominant-side limbs affected; 12 patients in the intervention group and 14 patients in the control group had nondominant-side limbs affected. In terms of post-stroke pain, 34 patients experienced no pain and 28 patients experienced pain. To be specific, 16 patients in the intervention group and 18 patients in the control group experienced no pain; 15 patients in the intervention group and 13 patients in the control group experienced post-stroke pain. Functional status was measured by the Barthel Index. The overall average score was 87.7 ± 18 scores. The average score in the intervention group and the control group was 86.2 ± 17.6 scores and 89.1 ± 18.5 scores, respectively. There was no significant difference between the 2 groups in terms of demographic data and stroke disease characteristics before the start of the study (Table 1).

**Table 1:**
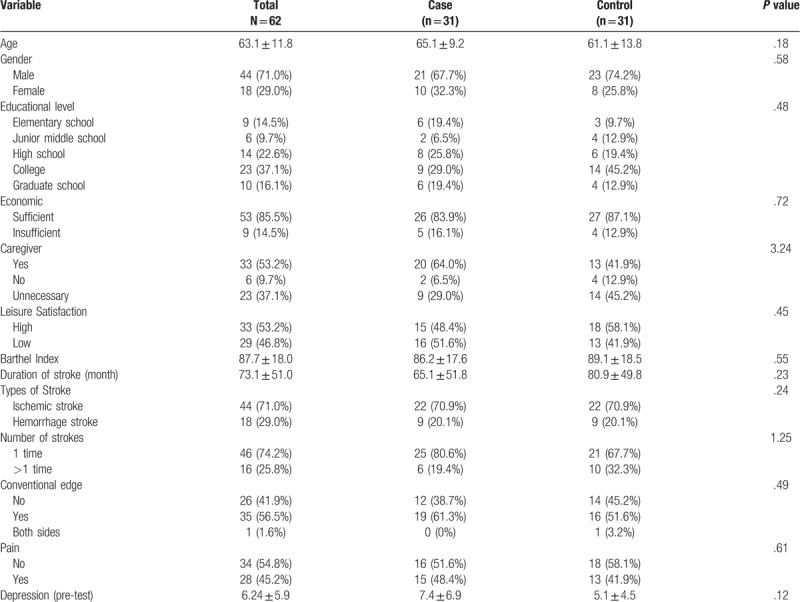
Basic demographics and disease characteristics.

In the intervention group that received social support and health education, there was a statistically significant difference in mean depression score measured at the 1st and 2nd weeks; the difference was also significant between the 4th week (6.03 ± 4.61), 8th week (5.29 ± 4.33), 12th week (6.00 ± 5.05), and pre-test score (7.41 ± 6.88), (all *P* < .05). After 4 weeks of social support and health education, the intervention group exhibited a significant difference in depression scores, while the control group exhibited no statistically significant changes. From the start of the study to the 8th week, the difference between the 1st and 2nd weeks and the difference between the 1st and 4th week were not statistically significant in the intervention group and the control group. After 8 weeks of continuous intervention, the two groups exhibited a statistically significant difference between the 1st and 8th weeks (Table 2).

**Table 2:**
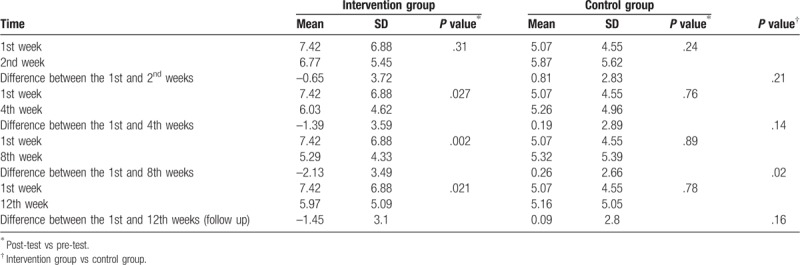
Depression score between intervention and control group.

Changes in depression scores (compared with that in the first week) were associated with economic status, duration of stroke, and overall satisfaction. In particular, the changes in depression scores were positively correlated with stroke duration and negatively correlated with overall satisfaction in the intervention group. The changes were not statistically correlated with age and educational attainment (Table 3).

**Table 3:**
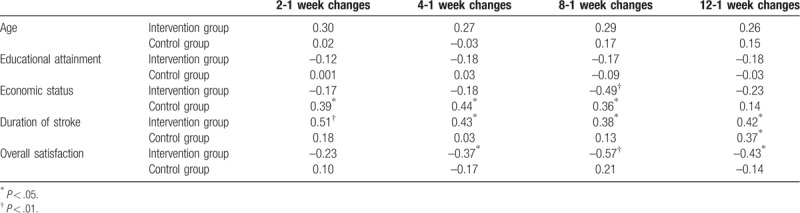
Spearman correlation analysis of depression score changes in intervention and control group.

After significantly correlated variables were incorporated into a GEE model, the depression score in the intervention group with social support and health education was found to be 1.24 points (*P* < .001) less than that in the control group. The depression score measured in the 2nd week was used as a reference. In the intervention group, the depression score measured in the 4th week was 0.76 points (*P* = .15) less than that measured in the 2nd week, and the depression score measured in the 8th week was 0.68 points (*P* = .15) less than that measured in the 2nd week. Thus, there was significant difference at different times in the intervention group. The mean depression score of patients with caregivers was 0.18 points (*P* = .61) less than that of patients without caregivers; the mean depression score of cash-strapped patients was 0.82 points (*P* = .13) higher than that of patients in a relatively secure financial position; the mean depression score of patients with pain was 0.71 points (*P* = .07) higher than that of patients without pain; the mean depression score of patients unsatisfied with their leisure life was 1.06 points (*P* = .02) higher than that of patients satisfied with their leisure life. Stroke duration 5 to 10 years (variable) was used a reference group. In the intervention group, the depression score of patients with less than 3 years of stroke duration was 0.24 points less than that in the reference group, suggesting no significant difference (*P* = .62); the depression score of patients with 3 to 5 years of stroke duration was 1.38 points less than that in the reference group, suggesting significant difference (*P* = .02); the depression score of patients with over 10 years of stroke duration was 0.84 points higher than that in the reference group, suggesting no significant difference (*P* = .1) (Table 4).

**Table 4:**
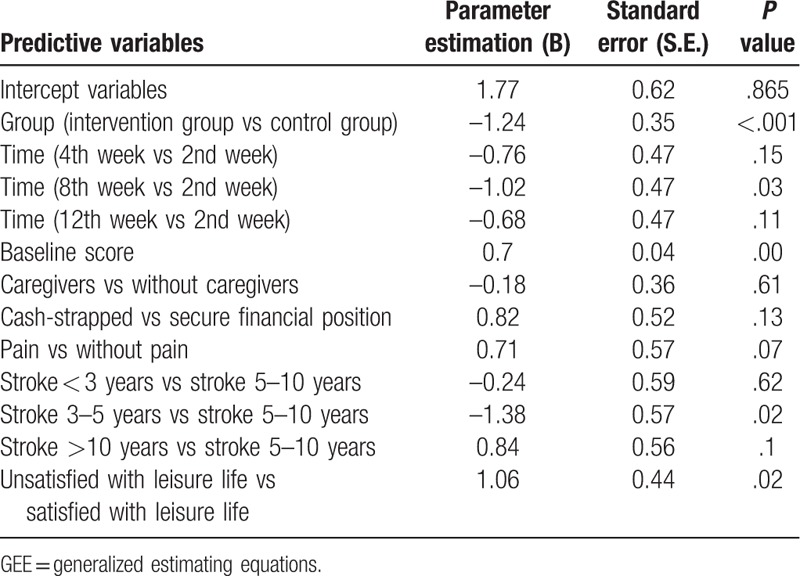
GEE model of depression score changes.

## 4 Discussion

Six months after stroke, 33.6% of the patients experienced more than 1 type of pain that had moderate or severe impact on their lives. Patients with severe depression had more chronic pain in the back and neck than those without pain.^[11–14]^ In the present study, the depression score of patients with pain was higher than that of patients without pain, but there was no significant difference although the depression scores were associated with pain. Therefore, if patients have both depressive symptoms and pain, we should first act to alleviate pain, which may help mitigate their depression. If patients have psychological stress and social phobia due to language disorder, we should encourage them to participate in leisure activities, which may help improve their physiological and psychological health and thus relieve depression.^[15]^ In a previous study, participation in leisure activities was found to be negatively correlated with depression in the elderly, and the higher frequency of activity participation, the lower degree of depression symptoms.^[8]^ This is consistent with the finding in the present study that the depression score of patients unsatisfied with their leisure life was higher than that of patients satisfied with their leisure life. Therefore, home activities and leisurely rehabilitation activities may be arranged for patients unsatisfied with their leisure life, so as to improve personal pleasure and satisfaction with activity participation, thereby improving their physiological and psychological health. In a previous study, researchers suggested preventive intervention for people with severe disability, a history of depression, cognitive impairment, stroke severity, and anxiety, or solitary people without caregivers in order to improve rehabilitation outcomes and their quality of life.^[2]^ In the present study, there was significant difference in depression scores between patients with caregivers and those without caregivers, which is consistent with previous findings reported. Stroke patients with caregivers exhibited a lower change in depression scores than those without caregivers. Thus, stroke patients without caregivers should first be provided with social welfare measures or referred to home care institutions. The results of our study suggest that depression can only be solved by first addressing the problem of caregivers.

In a randomized controlled empirical study, it was found that non-drug psychosocial intervention therapy may improve postpartum depression and psychosocial support had a short-term effect, but there is no evidence of a long-term effect.^[16]^ Other researchers reported that psychotherapy was conducted at least 12 to 20 times on patients with PSD, but the therapy was often abandoned due to language disorders, and the effect of entire execution was determined.^[17]^ In the present study, 16 interventions were carried out in 8 weeks, and significant differences were found in depression scores and depression tendency. After long-term follow-up of PSD and social support, we found that patients may need different types of social support at different times. Practical caring skills are needed at the early stages, while psychological needs are dominant at later stages. If a high degree of social support is provided, patients’ physical and psychosocial functions are better.^[5,17]^ Social support was negatively correlated with depression in patients with infarctive stroke, and informational support from family, relatives, and friends was reduced.^[18]^ In the present study, the social support and health education provided by functional therapists may give emotional and informational support to meet the physiological and psychological needs of stroke patients.

The subjects in this study were limited to patients in a medical center; the sample size was not large enough to be a representative sample; and the conclusion of the study may be not applicable to all chronic stroke patients. Patients with severe language disorders or cognitive impairments after stoke were not included in the study, and the number of patients with PSD may be underestimated. We recommend consulting primary caregivers or family members about the behavior or emotions of individual patients at home, so as to understand different emotions of patients with different post-stroke severity and provide the right support and care.

## 5 Conclusion

Continuous attention of clinical rehabilitation-related personnel to psychological health helps enhance the physical and mental well-being of stroke patients and improve their rehabilitation effect and quality of life. It was found that, in addition to 8-week continuous rehabilitation therapy twice a week, 10 additional minutes of emotional/informational support by functional therapists as required may have positive effects on relieving the depression of stroke patients. The results of the present study may be used as a reference for clinical practice and research on alleviating depression in stroke patients.

## Acknowledgments

The Chinese version of this article was accepted in 2017 for publication. We would like to contribute an English version of the article to English journals, which will be helpful to reach more audience and benefit more people. We hope the above information facilitates understanding of the work being submitted.

## Author contributions

**Conceptualization:** Fu-Huang Lin, Feng-Mei Shih, Chi-Ming Chu.

**Data curation:** Feng-Mei Shih.

**Formal analysis:** Fu-Huang Lin, Chi-Ming Chu.

**Investigation:** Feng-Mei Shih.

**Methodology:** Fu-Huang Lin, Daphne Ng Yih, Feng-Mei Shih, Chi-Ming Chu.

**Software:** Fu-Huang Lin, Daphne Ng Yih, Feng-Mei Shih.

**Supervision:** Fu-Huang Lin, Chi-Ming Chu.

**Validation:** Fu-Huang Lin.

**Writing – original draft:** Fu-Huang Lin, Feng-Mei Shih.

**Writing – review & editing:** Fu-Huang Lin, Daphne Ng Yih, Feng-Mei Shih, Chi-Ming Chu.
